# Optical absorption spectra and corresponding *in vivo* photoacoustic visualization of exposed peripheral nerves

**DOI:** 10.1117/1.JBO.28.9.097001

**Published:** 2023-09-04

**Authors:** Michelle T. Graham, Arunima Sharma, William M. Padovano, Visakha Suresh, Arlene Chiu, Susanna M. Thon, Sami Tuffaha, Muyinatu A. Lediju Bell

**Affiliations:** a Johns Hopkins University, Department of Electrical and Computer Engineering, Baltimore, Maryland, United States; b Johns Hopkins School of Medicine, Department of Plastic and Reconstructive Surgery, Baltimore, Maryland, United States; c Johns Hopkins University, Department of Biomedical Engineering, Baltimore, Maryland, United States; d Johns Hopkins University, Department of Computer Science, Baltimore, Maryland, United States

**Keywords:** photoacoustic imaging, multispectral, spectrophotometer, intraoperative imaging, peripheral nerve surgery, nerve injury, surgical guidance

## Abstract

**Significance:**

Multispectral photoacoustic imaging has the potential to identify lipid-rich, myelinated nerve tissue in an interventional or surgical setting (e.g., to guide intraoperative decisions when exposing a nerve during reconstructive surgery by limiting operations to nerves needing repair, with no impact to healthy or regenerating nerves). Lipids have two optical absorption peaks within the NIR-II and NIR-III windows (i.e., 1000 to 1350 nm and 1550 to 1870 nm wavelength ranges, respectively) which can be exploited to obtain photoacoustic images. However, nerve visualization within the NIR-III window is more desirable due to higher lipid absorption peaks and a corresponding valley in the optical absorption of water.

**Aim:**

We present the first known optical absorption characterizations, photoacoustic spectral demonstrations, and histological validations to support *in vivo* photoacoustic nerve imaging in the NIR-III window.

**Approach:**

Four *in vivo* swine peripheral nerves were excised, and the optical absorption spectra of these fresh *ex vivo* nerves were characterized at wavelengths spanning 800 to 1880 nm, to provide the first known nerve optical absorbance spectra and to enable photoacoustic amplitude spectra characterization with the most optimal wavelength range. Prior to excision, the latter two of the four nerves were surrounded by aqueous, lipid-free, agarose blocks (i.e., 3% w/v agarose) to enhance acoustic coupling during *in vivo* multispectral photoacoustic imaging using the optimal NIR-III wavelengths (i.e., 1630 to 1850 nm) identified in the *ex vivo* studies.

**Results:**

There was a verified characteristic lipid absorption peak at 1725 nm for each *ex vivo* nerve. Results additionally suggest that the 1630 to 1850 nm wavelength range can successfully visualize and differentiate lipid-rich nerves from surrounding water-containing and lipid-deficient tissues and materials.

**Conclusions:**

Photoacoustic imaging using the optimal wavelengths identified and demonstrated for nerves holds promise for detection of myelination in exposed and isolated nerve tissue during a nerve repair surgery, with possible future implications for other surgeries and other optics-based technologies.

## 1 Introduction

There are multiple potential causes of peripheral nerve injury, including trauma, intraoperative procedures, and iatrogenic injury. For example, limb trauma patients can suffer from chronic peripheral nerve injury, the majority of which are caused by motor vehicle accidents.^1^^–^^3^ Intraoperatively, nerves can be accidentally injured by mechanisms, such as direct transection (e.g., being mistaken as other tissues), stretch or compression (e.g., a retractor keeping the surgical area exposed, placement of orthopedic implants, improper patient positioning), and thermal damage (e.g., nearby coagulation).^4^^–^^7^ In addition, patients receiving nerve blockades can also suffer from nerve damage if the anesthetic needle is not correctly placed relative to the targeted peripheral nerves.^8^^,^^9^ Over time, these various groups of patients can suffer from symptoms such as chronic motor dysfunction, sensory dysfunction, decreased dexterity, or pain, and they are eventually referred to specialized centers to diagnose and treat nerve injury,^1^^,^^2^^,^^10^^–^^12^ where treatment often includes surgery to expose, isolate, and repair the nerve.

To prevent or treat nerve injury, surgeons, anesthesiologists, and other clinicians primarily rely on known anatomical relationships and electrodiagnostics studies. However, known anatomical relationships become distorted when anatomy is disrupted during a surgery or when operating on diseased or damaged tissues. Electrodiagnostic studies include electromyography (EMG) and nerve conduction studies (NCS), which monitor skeletal muscle activity and nerve conduction ability, respectively.^13^ Combining EMG and NCS with the stimulation of nerve tissue provides an indication of general nerve proximity and general nerve function, but it suffers from imprecise assessments of nerve localization^14^ and nerve architecture.^15^ In addition, EMG is restricted to identifying only a subset of nerves (i.e., EMG is ineffective at locating sensory nerves, and it is similarly ineffective at locating motor nerves that may not stimulate normally due to prior trauma or intraoperative paralytics).^16^^–^^18^

Imaging techniques are increasingly being explored with promising potential to address the above-stated challenges. For example, ultrasound imaging can be used in surgical planning to preoperatively map nerve locations and determine a suitable surgical pathway.^19^^,^^20^ In an interventional or intraoperative setting, ultrasound imaging has the potential to offer real-time visualization of nerves and their proximity to needles^8^ or other foreign bodies, such as orthopedic implants.^21^ However, nerves can be confused with the similar appearance of nearby soft tissue structures in ultrasound images,^22^ and changes in local anatomy can alter the expected nerve appearance.^11^ In addition, needle tips can be difficult to identify in ultrasound images.^23^ The success of ultrasound imaging is also subject to operator skill and expertise.^24^^,^^25^ Magnetic resonance imaging (MRI) is an alternative option to provide excellent soft tissue contrast and nearby anatomical landmarks with less dependence on operator performance.^11^ However, MRI is expensive, time-consuming to implement, and unsafe to operate in the presence of ferromagnetic metallic devices or implants.^26^ Near-infrared fluorescence image-guidance has also been proposed, but it requires the injection of contrast agents.^27^

Photoacoustic imaging is a promising alternative to existing options for interventional localization of nerve tissue during nerve block injections, routine surgeries, and nerve repair surgeries.^28^^–^^36^ To implement photoacoustic imaging, pulsed light is transmitted and selectively absorbed based on the inherent optical properties of endogenous chromophores or metallic needle tips and then converted to acoustic energy, with an ultrasound transducer receiving the resulting signals.^37^^,^^38^ In nerve tissue, which contains axons wrapped in insulating myelin sheaths and bundled together by collagenous connective tissue and fatty deposits,^10^ the lipids within the myelin sheaths and fat cells preferentially absorb transmitted light to achieve photoacoustic images of nerves.^28^^,^^29^

Photoacoustic imaging of nerves is challenged by the comparable optical absorption properties of lipids and surrounding tissue at singular wavelengths.^39^^–^^43^ These similar properties reduce target contrast and impede photoacoustic monitoring of nerves. As a possible solution, lipids are known to have unique optical absorption spectra as a function of multiple illuminating wavelengths, which can be measured by the relative amplitude of the photoacoustic signal at each wavelength (i.e., the photoacoustic amplitude spectra).

Excitation wavelengths that exploit the optical absorption peaks of lipids at 1210 and 1730 nm were previously identified as optimal for photoacoustic imaging of lipid-rich nerve tissue.^44^^,^^45^ These wavelengths reside in the second near infrared optical window of ∼1000 to 1350 nm, hereafter referred to as NIR-II,^44^^,^^46^^–^^49^ and in the third near infrared optical window of ∼1550 to 1870 nm, hereafter referred to as NIR-III.^44^^,^^46^^,^^49^ Using these wavelength ranges, the photoacoustic amplitude spectra measured from *ex vivo* peripheral nerve tissues were compared with the optical absorption of lipid samples, such as subcutaneous fat. Agreement between photoacoustic amplitude spectra and lipid optical absorption spectra confirmed both the identification of the lipids in nerve tissue^28^ and the differentiation of nerve tissue from other structures, such as collagenous tendons^29^^,^^34^ and hemoglobin-rich blood vessels.^31^

Li et al.^36^ advanced previous studies and demonstrated the use of multispectral photoacoustic imaging to differentiate an *in vivo* femoral nerve from the femoral artery in a mouse model using a subset of NIR-II wavelengths (i.e., 1100 to 1250 nm). Wang et al.^35^ compared the photoacoustic signal acquired with one NIR-II wavelength (i.e., 1210 nm) with that acquired with a subset of NIR-III wavelengths (i.e., 1600 to 1850 nm) and demonstrated that the photoacoustic signal from *ex vivo* intramuscular goat fat was five times greater when targeting the lipid absorption peak at 1730 nm, rather than the lipid absorption peak at 1210 nm. In addition, the 1730 nm lipid absorption peak resides in a valley of the absorption spectra of water, thus providing a biological optical window to target the lipids within nerve tissue.

The success of these previous photoacoustic nerve imaging approaches^28^^,^^29^^,^^31^^,^^34^^–^^36^ required two essential components. First, the range of excitation wavelengths was strategically chosen to produce photoacoustic amplitude spectra of nerves that are distinguishable from the photoacoustic amplitude spectra of surrounding tissues. Second, reference lipid optical absorption spectra were available to confirm which measured photoacoustic amplitude spectra originated from nerve tissue. However, no previous work has performed *in vivo* photoacoustic nerve imaging with wavelengths that leverage the 1730 nm lipid peak (which was identified as preferred over the 1210 nm peak in a previous *ex vivo* study^35^). In addition, the optical absorption spectra of isolated lipids^40^^,^^43^^,^^50^^,^^51^ were previously used to determine that the likely source of the visualized signals was nerve tissue. We hypothesize that characterizing the optical absorption of myelinated nerve tissue will improve both wavelength selection and nerve tissue identification with photoacoustic imaging, particularly when compared with the current information available with the optical absorption spectra of isolated lipids.

This paper presents the first known characterization of the optical absorbance spectra of fresh myelinated nerve samples using a wide spectrum of wavelengths (i.e., 800 to 1880, which span the NIR-I, NIR-II, and NIR-III optical windows). We also present the first known *in vivo* visualization and corresponding optical absorption characterization of nerve contents using multispectral photoacoustic imaging and the identified optimal wavelengths for composite nerve tissue (i.e., wavelengths 1630 to 1850 nm in the NIR-III optical window). These optimal wavelengths for photoacoustic imaging of composite nerve tissue are hereafter defined as the “NIR-III nerve window.” In addition, the structural and anatomical content of the imaged *in vivo* nerve tissue was confirmed with histology. We focus on nerves in isolation, rather than nerves surrounded by competing photoabsorbers because it is important to understand the properties of nerve tissue alone, prior to understanding interactions and differentiation from surrounding media. In addition, a single isolated nerve provides an excellent ground truth for *in vivo* investigations. These fundamental contributions are intended to build the scientific foundation necessary to benefit multiple optics-based nerve visualization and assessment applications. The remainder of this paper is organized as follows. Section 2 describes the experimental methods employed to achieve our primary contributions. Section 3 presents the corresponding results, Sec. 4 discusses the implications of our results, and Sec. 5 concludes the paper.

## 2 Methods

### 2.1 Nerve Samples

A total of eight peripheral nerve samples were harvested from three Yorkshire swine (Archer Farms, Maryland), hereafter referred to as swine I, swine II, and swine III. These nerve samples consisted of one regenerated median nerve (i.e., 1 year after injury and repair) dissected into two samples from swine I (i.e., two median nerve samples) and one ulnar nerve dissected into two samples each from swine I, swine II, and swine III (i.e., six control ulnar nerve samples). Of the two regenerated nerve samples, one was used for spectroscopic absorbance measurements and one was used for histological analysis. Of the six control nerve samples, three (i.e., one from each swine) were used for spectroscopic absorbance measurements, and the remaining three were used for histological validation. The samples from swine II and III were available for these measurements prior to those from swine I, which was imaged after the availability of these measurements. Additional details regarding spectroscopic absorbance measurements, *in vivo* photoacoustic imaging, and histological validation are described in Secs. 2.2, 2.3, and 2.4, respectively. The regenerated nerve samples represent nerve anatomy that deviates from normal (e.g., in patients recovering from nervous system diseases or nerve injuries), and the control samples represent normal.

### 2.2 Spectroscopic Absorbance Measurements

**Fig. 1:**
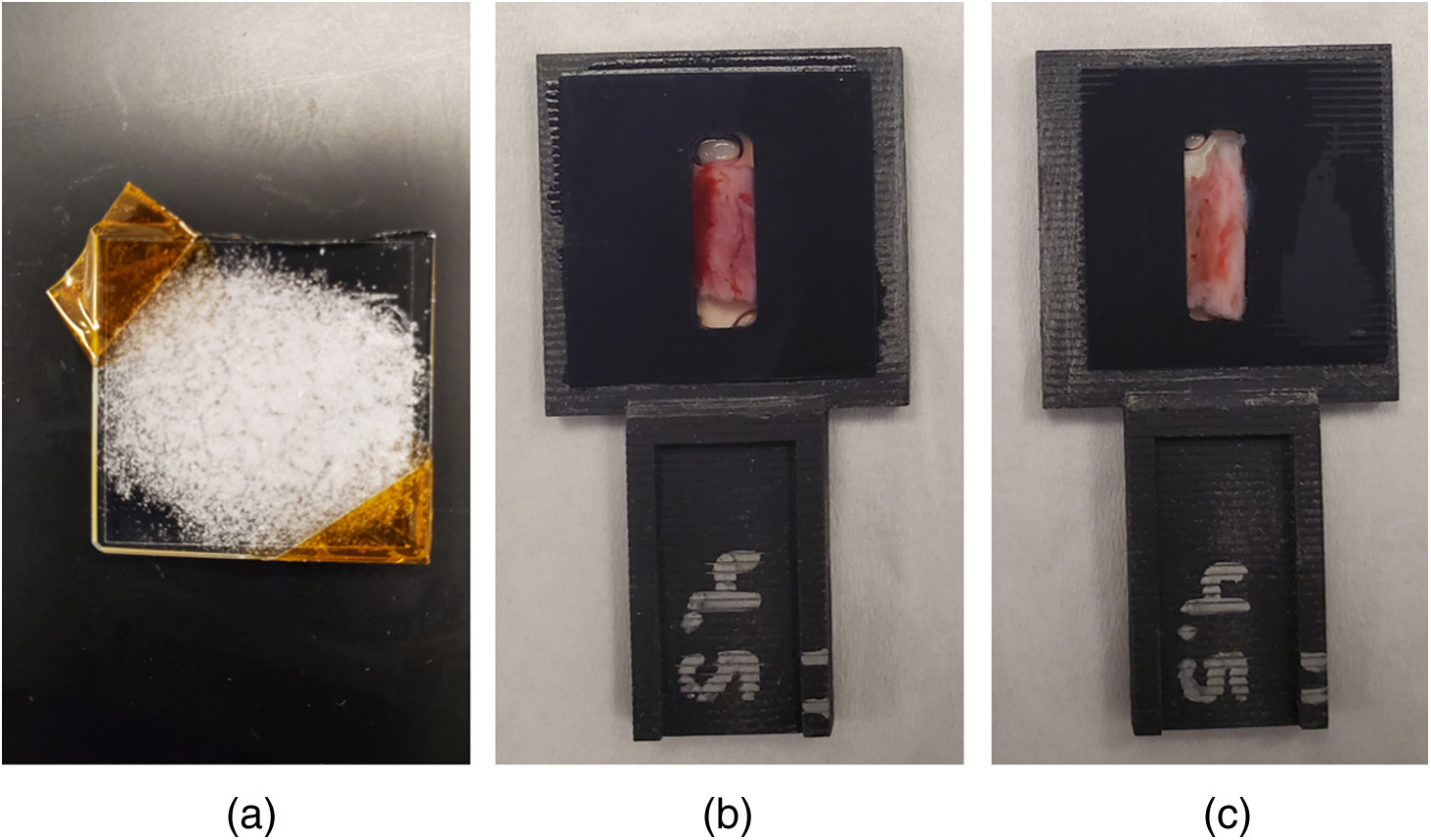
Samples mounted for placement in the DRA centermount including (a) powdered cholesterol between two glass slides and (b) median and (c) ulnar nerves mounted in the 3D-printed sample holders.

Absorbance measurements were obtained with a Cary 5000 dual beam spectrophotometer and external diffuse reflectance accessory (DRA) from Agilent Technologies (DRA-2500, Santa Clara, California) for the following five samples: (1) powdered cholesterol (C8667, Sigma-Aldrich, St. Louis, Missouri); (2) 3%w/v agarose (A6013, Sigma-Aldrich, St. Louis, Missouri), (3) phosphate buffered saline (PBS), which is a water-based salt solution (10010023, Thermo Fischer Scientific, Waltham, Massachusetts); (4) three fresh swine ulnar nerves; and (5) one fresh swine median nerve. In the context of this paper, fresh is defined as spectroscopic absorbance measurements being performed ≤24 h after harvesting. Figure 1 shows examples of samples mounted for placement in the DRA centermount port. The cholesterol sample (representing the primary lipid in the myelin sheath of nerves^52^^–^^55^) was deposited between two glass slides [Fig. 1(a)]. This cholesterol sample was included to provide a baseline reference point for our nerve measurements using the same equipment and process. To mount the nerves for spectroscopic analysis, a 3D-printed sample holder was designed with a rectangular cavity to hold the nerve in place during data acquisition [Figs. 1(b) and 1(c)]. PBS was added to the rectangular cavity to minimize air interfaces, and coverslips were attached to each side of the nerve sample holder. The coverslips secured the nerve inside the holder and limited dehydration of the tissue. To obtain the PBS absorbance measurement (which represents the absorbance of water), the rectangular cavity of the sample holder was filled with PBS and secured with two coverslips. To obtain the agarose absorbance measurements, the agarose sample was sliced from an agarose block and adhered to a coverslip on a rectangular slit sample holder.

Prior to sample measurements, 100% and 0% absorbance baselines were obtained. For each sample, the 100% absorbance baseline was obtained by completely blocking the beam path into the DRA. The 0% absorbance baselines for each sample were obtained using an appropriate “blank” (i.e., two bare glass slides for cholesterol and an empty rectangular slit sample holder for PBS, agarose, and nerve). Each sample was placed in the DRA centermount at an incident angle of 5 deg. The wavelength measurement range was 800 to 1800 nm in 1 nm increments (which spanned the NIR-I, NIR-II, and NIR-III optical windows), and the averaging time was 0.1 s per wavelength.

### 2.3 *In Vivo* Photoacoustic Imaging

Our photoacoustic imaging system consists of a Phocus Mobile system (Opotek, Carlsbad, California), an E-CUBE 12R ultrasound scanner (Alpinion Medical Systems, Seoul, South Korea), and an Alpinion L8-17 ultrasound transducer. In the Phocus Mobile system, a Q-switched Nd:YAG laser, operating at 10 Hz with a 5 ns pulse width, was coupled to an optical parametric oscillator (OPO). The OPO was operated at idler wavelengths in the NIR-III nerve window, specifically 1630 to 1850 nm in 5 nm increments. This wavelength range was chosen based on the lipid absorption peak reported in the existing literature^35^ and the spectroscopic absorbance measurements obtained from nerves of swine II and III. A motorized variable attenuator (MVA) was placed in the beam path between the OPO output and an optical fiber bundle, with 3.9 mm-diameter bifurcated ends (Armadillo SIA, Sunnyvale, California). The MVA was calibrated to ensure that the bifurcated ends of the optical fiber bundle emitted a mean total energy of 4.2  mJ/pulse at each wavelength in the NIR-III nerve window (mean fluence of $∼18\;\mathrm{mJ}/{\mathrm{cm}}^{2}$).

To determine the possible agreement between the amplitude of photoacoustic signals achieved in an *in vivo* setting and the spectroscopic absorbance measurements for nerve tissue, the ulnar (control) and median (regenerated) nerve samples from swine I (see Sec. 2.1) were imaged with our photoacoustic imaging system prior to resection. The regenerated median nerve model involved sharp transection of the median nerve with immediate tension-free microsurgical repair using 8-0 nylon epineurial sutures followed by a recovery period of one year. To perform imaging, the ulnar and median nerves in the proximal forelimb were first exposed, and then each underwent epineurectomy to remove the influence of excess connective tissue on the photoacoustic measurements of the nerve tissue.

**Fig. 2:**
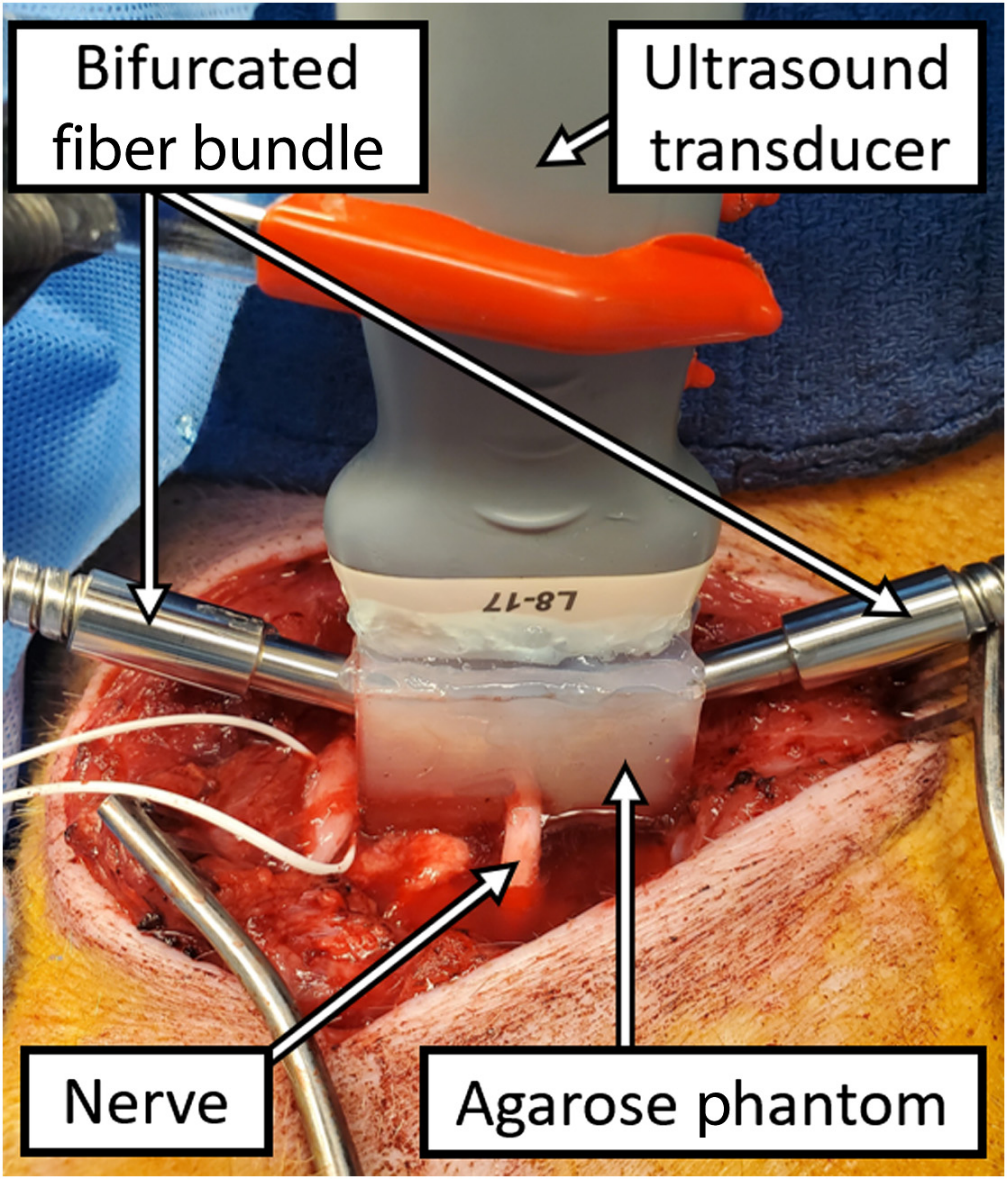
Experimental setup for *in vivo* photoacoustic imaging, including bifurcated fiber bundles secured in a custom-molded agarose block to bilaterally illuminate the nerve, with an ultrasound transducer positioned to image a circular cross section of the illuminated nerve.

To provide acoustic coupling for photoacoustic imaging, a custom 3% w/v agarose block was placed to surround each nerve, and the bifurcated optical fiber bundle described above was inserted into cavities within the agarose block, as shown in Fig. 2. In addition to providing acoustic coupling, the custom agarose block also secured the relative positions of the nerve and imaging components for the duration of the experiment and positioned the nerve at a reasonable distance of 1.75 cm from the surface of the ultrasound transducer. The ultrasound transducer was placed orthogonal to the surface containing the opposing fiber bundle cavities within the agarose block to image a circular cross section of the nerve. The imaged nerves were then resected after euthanasia to create the four samples from swine I described in Sec. 2.1. These procedures were approved by the Johns Hopkins University Animal Care and Use Committee (Protocol SW20M73).

Co-registered ultrasound and photoacoustic images were generated from channel data with delay-and-sum (DAS) beamforming. To reduce the impact of pulse-to-pulse energy variations, 10 beamformed images per wavelength were averaged to form a single normalized photoacoustic image for spectral analysis at each wavelength. To create nerve and background regions of interest (ROIs) for the spectral analysis, the normalized photoacoustic images acquired with 1230 and 1725 nm wavelengths were first thresholded to display signal amplitudes ≥0.15. Artifacts (i.e., signals surviving the threshold yet located outside of the nerve or agarose-fiber interface locations) were manually removed, the location of remaining signals were combined using the Boolean algebra logical OR operation, and the combined result was separated into nerve and background ROIs. The amplitude of each DAS image pixel within these ROIs was measured as a function of wavelength to generate photoacoustic amplitude spectra for the NIR-III nerve window.

To demonstrate the advantage of the NIR-III nerve window relative to NIR-II wavelengths, we additionally operated our photoacoustic system at the NIR-II wavelengths that we have access to with our system (i.e., 1230 to 1450 nm). Otherwise, the same system, *in vivo* setup, nerve locations on swine I, and ROIs described above were employed to report the amplitude of each DAS image pixel within the ROIs as a function of NIR-II wavelengths.

### 2.4 Histological Validation

Toluidine blue staining of the resected nerve samples from swine I, II, and III was performed to provide ground truth anatomical and structural information of the nerve samples, including (1) confirming the presence of lipids in the nerve samples, (2) assessing the lipid distribution within the nerve samples, and (3) quantifying structural differences between nerve samples. In particular, the nerve samples were fixed for 48 h at 4°C in a solution of 2% glutaraldehyde, 3% paraformaldehyde, and 0.1 M PBS (pH 7.2). Samples were then post-fixed in 2% osmium tetroxide, dehydrated in ascending alcohol series, and embedded in resin prior to staining with 1% Toluidine blue. Representative slices were evaluated using a Zeiss Axioplan 2 microscope (Carl Zeiss Microscopy LLC, White Plains, New York), and images were captured using a Jenoptik ProgRes C5 camera (Jupiter, Florida) mounted to the microscope. At 100× magnification, a single nerve slice produced 4 to 14 non-overlapping micrograph segments. Micrograph segments (.tif files, 2580×1944  pixels, 15 MB) were imported into ImageJ (FIJI Package, version 2.0, NIH, Bethesda, Maryland) and analyzed by a blinded assessor.^56^^,^^57^ Histomorphometric assessment of nerve and myelin anatomy was performed by measuring the nerve density, fiber diameter, myelin thickness, and ratio of the axon diameter to the fiber diameter (i.e., G-ratio). The G-ratio indicates the degree of axon myelination, with a value of 1.0 indicating a completely unmyelinated axon.

## 3 Results

### 3.1 Optical Absorption Characterization with *Ex Vivo* Samples

**Fig. 3:**
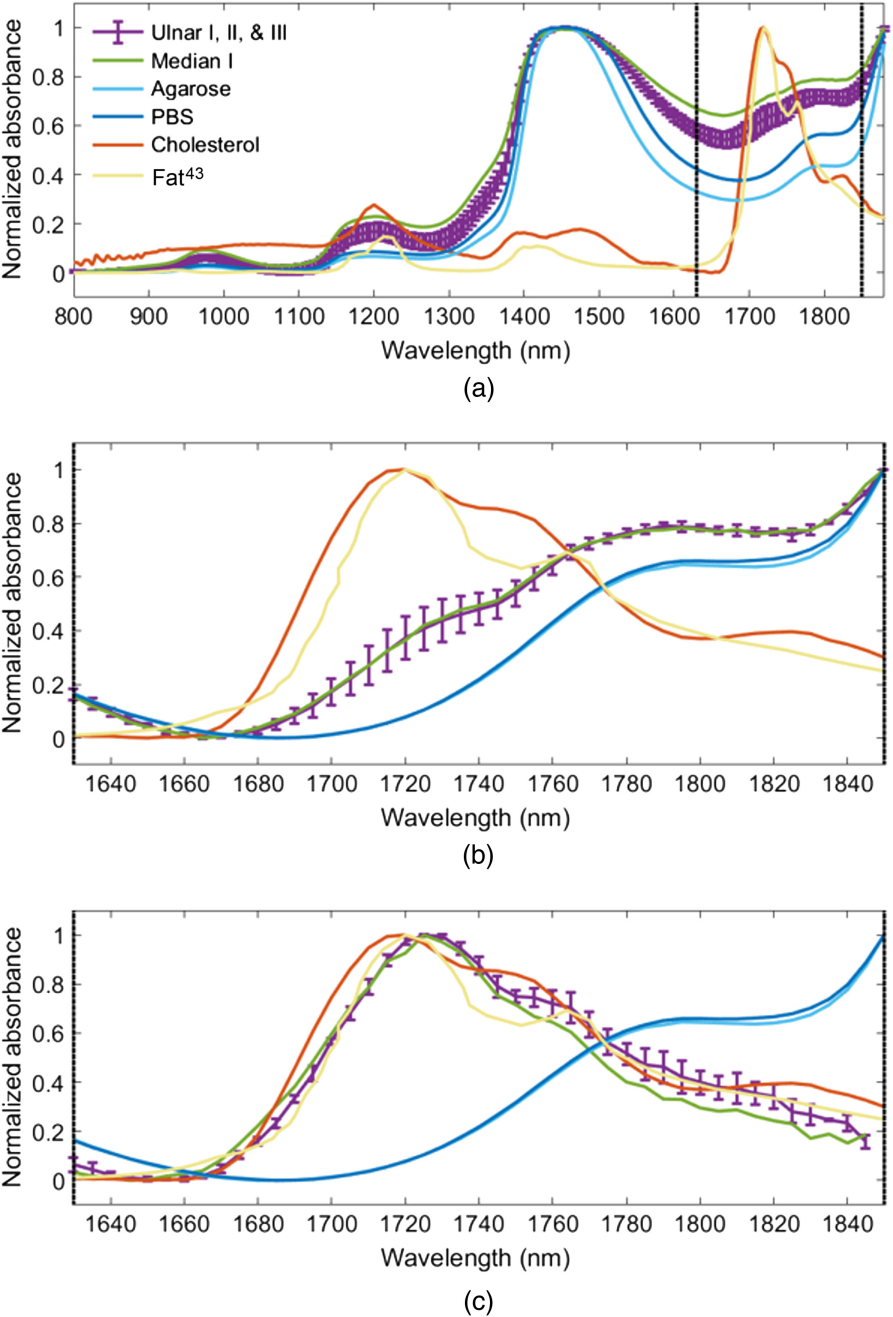
Absorbance measurements of three ulnar nerve samples (mean ± one standard deviation), one median nerve sample, and samples of powdered cholesterol, 3% w/v agarose, and PBS, shown for (a) 800 to 1880 nm wavelengths, (b) 1630 to 1850 nm wavelengths [indicated by vertical dashed lines in panels (a)–(c)], and (c) 1630–1850 nm wavelengths after the PBS absorbance spectrum was subtracted from each nerve measurement. In each case, the normalized optical absorption of fat was extracted from Ref. 43 and is shown for comparison.

Figure 3 shows spectroscopic optical absorbance measurements of the five samples noted in Sec. 2.2 (i.e., PBS, 3% w/v agarose, cholesterol, one median nerve, and the average ± one standard deviation of three ulnar nerve samples). The normalized optical absorption spectrum of fat, a lipid previously employed for comparison with nerve optical absorption,^51^ was obtained from Ref. 43 and plotted with the results in Fig. 3.

The absorbance measurements for a wide range of wavelengths measured by the spectrophotometer (i.e., 800 to 1800 nm) are available in Fig. 3(a). In the NIR-II range, cholesterol and nerve tissue each have an absorbance peak at 1210 nm, which is similar to that observed in fat^43^ and other isolated lipids.^40^^,^^50^ Agarose, PBS, and nerve tissue each have an absorbance peak at 1450 nm, which is similar to that of water.^41^^,^^58^ In the NIR-III range, cholesterol has a second absorbance peak at 1715 nm, and fat^43^ has absorbance peaks at 1720 and 1765 nm, respectively.

To better view the absorbance characteristics of the samples in the NIR-III nerve window [indicated by the dashed lines in Fig. 3(a)], Fig. 3(b) presents a zoomed and normalized version of Fig. 3(a). Although the agarose, PBS, and nerve samples have similar absorbance measurements in this window of wavelengths (i.e., 1680 to 1850 nm), the nerve absorbance measurements also deviate from that of the water-based samples (i.e., agarose and PBS) and do not contain an expected lipid absorbance peak at 1715 nm, as demonstrated in the cholesterol and fat measurements. Instead, nerve tissue and water-based agarose and PBS absorbance measurements each monotonically increase in the range of 1680 to 1790 nm and reach a plateau in the wavelength range 1790 to 1830 nm, suggesting that water is the dominating chromophore in nerve tissue.

To remove the dominating contribution of water from the nerve absorbance measurements, Fig. 3(c) shows the nerve absorbance spectra after the PBS absorbance spectrum was subtracted from each nerve measurement. To perform the subtraction, each measured absorbance spectra was first normalized by its maximum value and then scaled to an amplitude range of 0 to 1, and the normalized and scaled PBS absorbance spectrum was subtracted from each normalized and scaled nerve absorbance spectrum. This difference was scaled to an amplitude range of 0 to 1 to create the isolated nerve absorbance spectra shown in Fig. 3(c). These isolated nerve absorbance spectra have an absorbance peak at 1725 nm, which differs from the absorbance peak of fat^43^ and cholesterol by 5 and 10 nm, respectively. The isolated nerve absorbance spectra also have either a plateau or a decreased rate of change in the range of 1745 to 1765 nm, which is similar to that of fat^43^ and cholesterol in the same wavelength range. These similarities and differences highlight expected subtleties between the absorption spectra of various lipid-rich structures. The presence of the observed 1725 nm peak and surrounding absorption profile for composite nerve tissue also informs optimal wavelengths to employ when acquiring photoacoustic images of nerves. In addition, the results in Fig. 3 demonstrate that both water and lipid contribute to the absorbance spectra of the ulnar and median nerve samples.

### 3.2 *In Vivo* Photoacoustic Characterizations and Comparisons with *Ex Vivo* Spectrophotometer Results

**Fig. 4:**
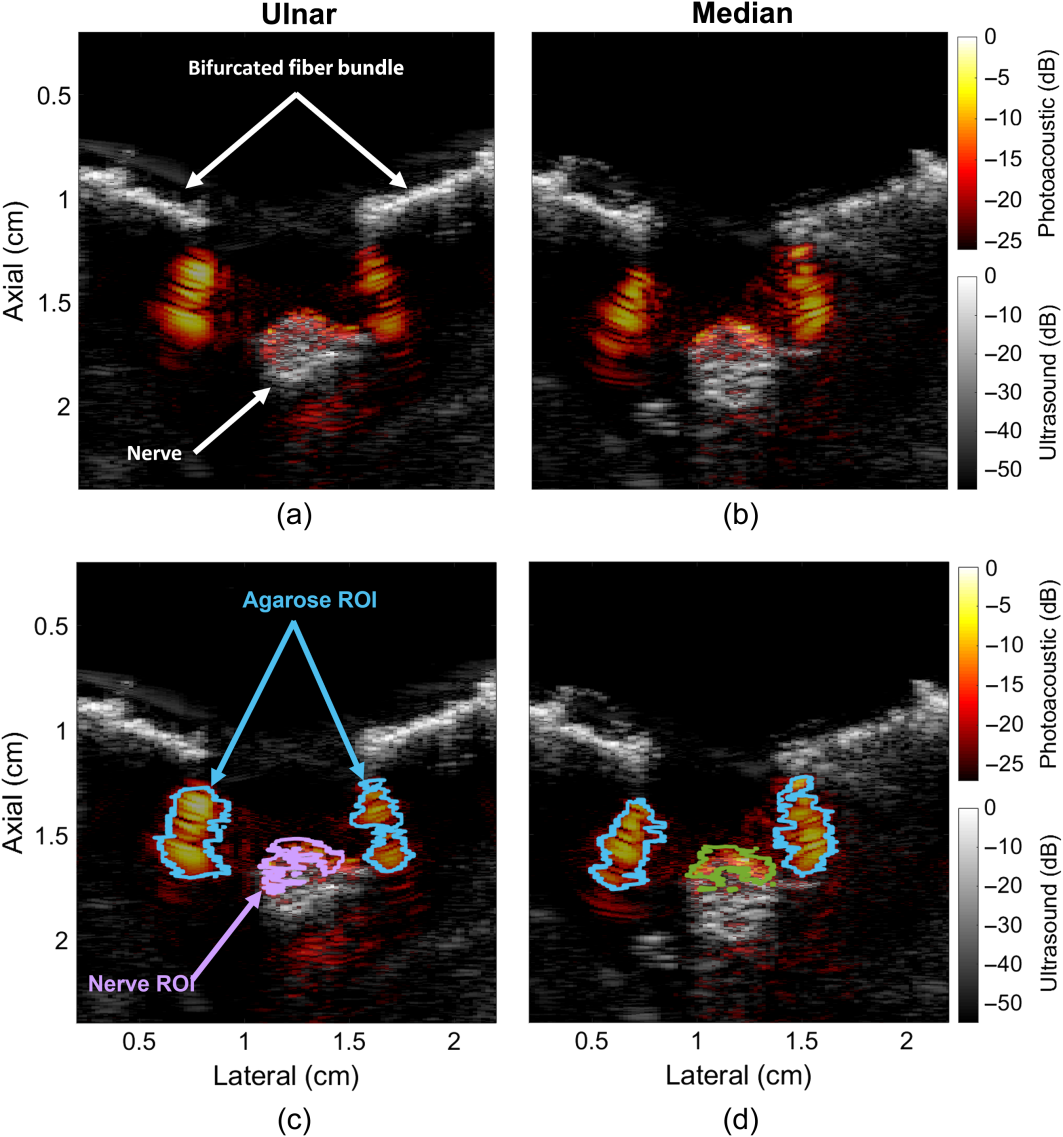
Photoacoustic images of the (a) ulnar and (b) median nerves from swine I (i.e., ulnar nerve I and median nerve I, respectively), illuminated with 1725 nm light and overlaid on co-registered ultrasound images. Replicated images of (c) ulnar nerve I and (d) median nerve I, showing outlines of the nerve and agarose ROIs, which were created as described in Sec. 2.3. Only the top of each fiber bundle is shown as the bright reflective structures (indicated by the arrows in the ultrasound image), whereas the interface between the full optical aperture of the fiber bundle tip and the agarose phantom is visible in the photoacoustic images, which is responsible for creating the agarose signal (indicated by the blue arrows). The lower amplitude photoacoustic signals located distal to the nerve are likely reflection artifacts.

Figure 4 shows co-registered photoacoustic and ultrasound images of the ulnar and median nerves from swine I when bilaterally illuminated with an optical wavelength of 1725 nm. Photoacoustic signals are primarily present in the superficial, superior area of the circular nerve cross section in Figs. 4(a) and 4(b). In addition, photoacoustic signals are observed immediately distal to the bifurcated light sources, which indicate photoacoustic signals originating from the agarose block. Figures 4(c) and 4(d) highlight the ROIs utilized to measure the photoacoustic amplitude spectra, with details on ROI selection available in Sec. 2.3.

**Fig. 5:**
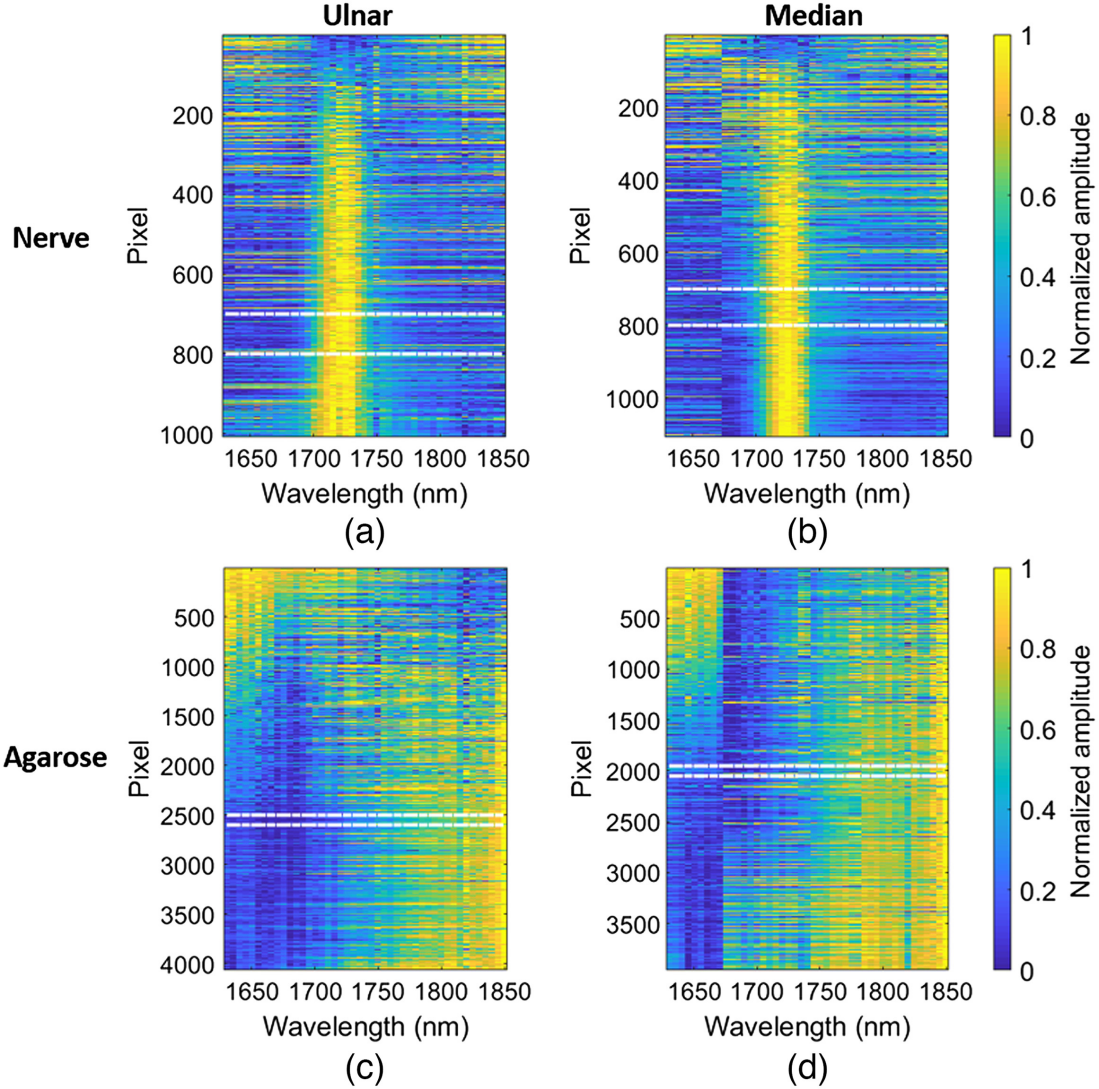
*In vivo* photoacoustic amplitude spectra of the samples computed for each pixel within the ROI for (a) ulnar nerve I, (b) median nerve I, (c) the agarose block used to image ulnar nerve I, and (d) the agarose block used to image median nerve I. The photoacoustic amplitude spectrum of each pixel is normalized between 0 and 1. Dashed white lines outline data extracted to create Fig. 6.

Figure 5 shows the measured photoacoustic amplitude spectra of each pixel within the ROIs shown in Figs. 4(c) and 4(d). The photoacoustic amplitude spectra of the ulnar and median nerve each have a peak at 1725 nm. In direct contrast, the photoacoustic amplitude spectra of the corresponding agarose blocks do not have a single characteristic peak in the NIR-III nerve window. Instead, the photoacoustic amplitude spectra of both agarose blocks show increased photoacoustic amplitude at the lower and upper boundaries of the NIR-III nerve window (i.e., 1630 to 1675 nm and 1740 to 1850 nm, respectively) with a valley in the 1675 to 1740 nm range. The white dashed lines in Fig. 5 (i.e., independent groupings of 100 pixels that maximized the dominant peak signal location and minimized the standard deviation outside of each dominant peak signal of interest) indicate regions utilized to plot photoacoustic amplitude spectra for direct comparison with spectrophotometer measurements.

**Fig. 6:**
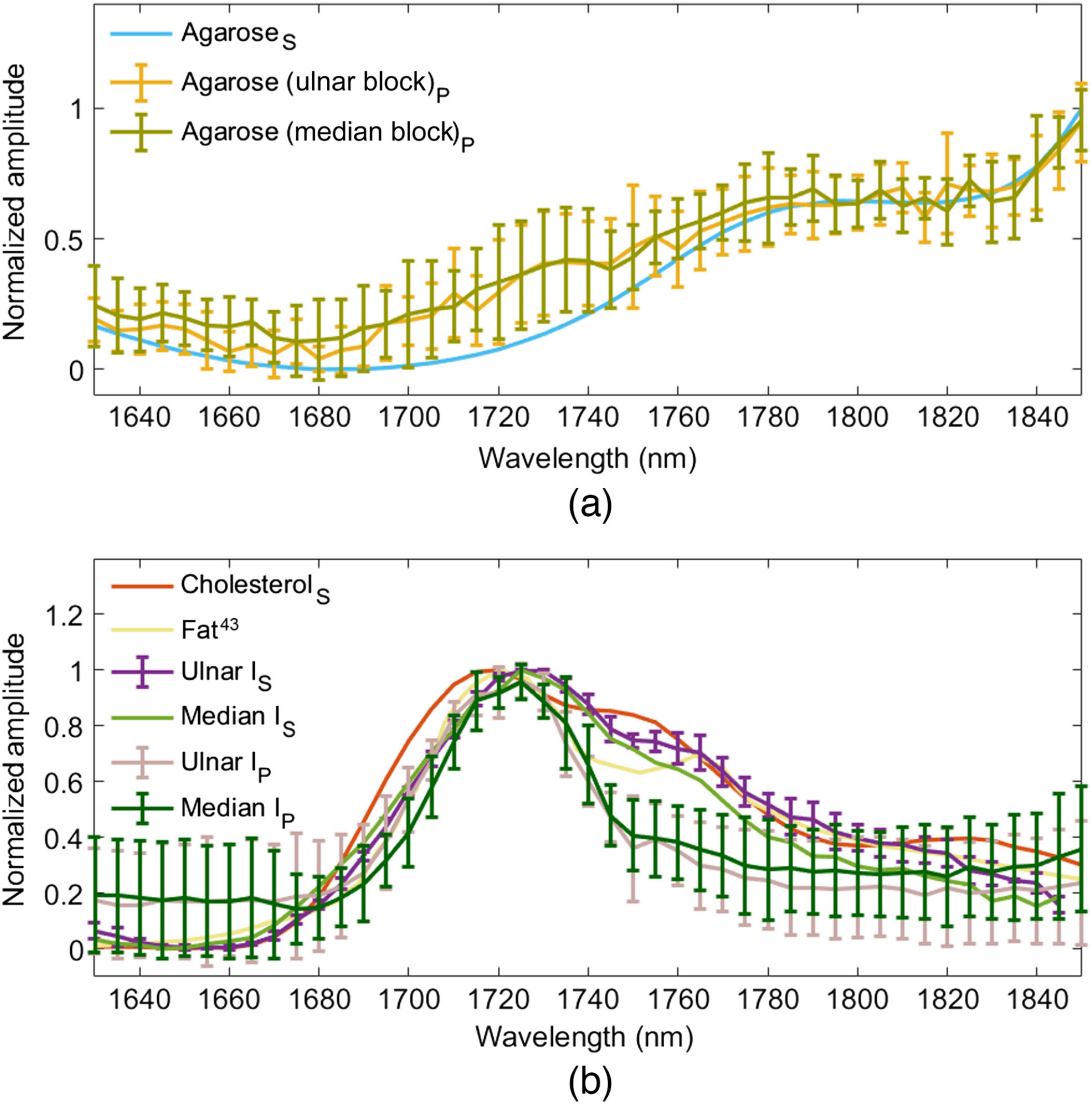
Comparison of absorbance spectra measured with the spectrophotometer (subscript S) and photoacoustic amplitude spectra (subscript P) for the (a) agarose blocks, (b) cholesterol, ulnar nerve, and median nerve. The mean ± one standard deviation of the absorbance spectra is shown for ulnar nerve I, II, and III. The mean ± one standard deviation of the photoacoustic amplitude spectra is shown for pixels between the horizontal dashed lines in Fig. 5.

Figure 6 directly compares the mean ± one standard deviation of the photoacoustic amplitude spectra residing within the horizontal dashed lines in Fig. 5 and the corresponding absorbance spectra replicated from Fig. 3. There is general agreement between the absorbance spectra and photoacoustic amplitude spectra of agarose in Fig. 6(a). In addition, the photoacoustic amplitude spectra of agarose are reproducible across the two manufactured agarose blocks. Figure 6(b) compares the photoacoustic amplitude spectra of the ulnar and median nerves with the absorbance measurements of the ulnar and median nerve samples after the PBS absorbance spectra were subtracted from each nerve measurement. The photoacoustic amplitude spectra of the ulnar and median nerves have a peak at 1725 nm, which agrees with the 1725 nm peak in the corresponding absorbance spectra. This peak differs from that of fat^43^ and cholesterol by 5 and 10 nm, respectively.

### 3.3 Comparisons of Photoacoustic Amplitude Spectra within NIR-II and NIR-III Wavelengths

**Fig. 7:**
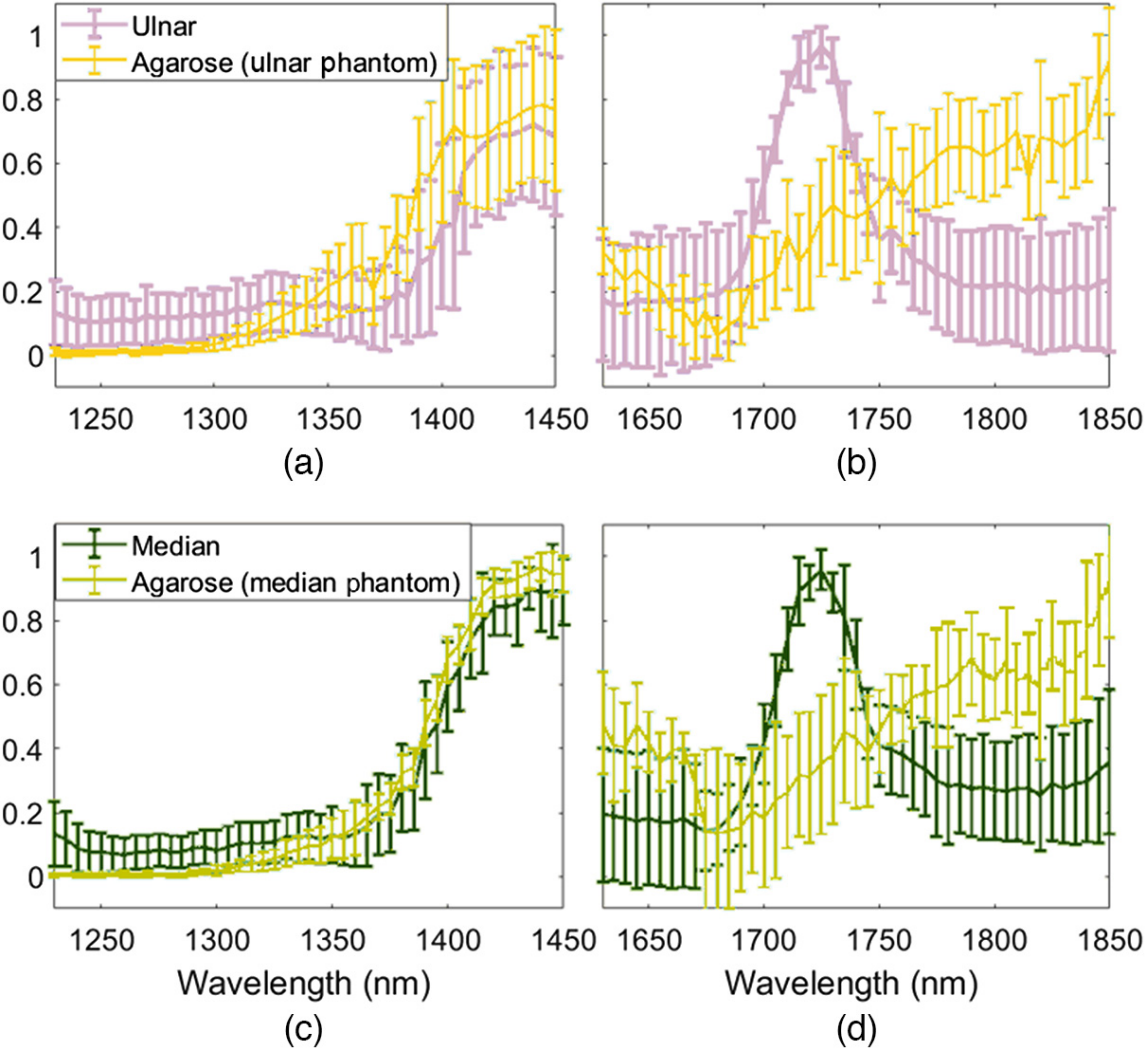
Comparison of photoacoustic amplitude spectra at NIR-II (1230 to 1450 nm) and NIR-III (1630 to 1850 nm) wavelengths, acquired from swine I. (a), (b) Ulnar nerve compared with agarose surrounding the ulnar nerve. (c), (d) Median nerve compared with agarose surrounding the median nerve. NIR-III results are duplicated from Fig. 6 (i.e., mean ± one standard deviation of the photoacoustic amplitude spectra) with agaorose and nerve results overlapped on the same plots.

Figure 7 shows a comparison of the NIR-II and NIR-III photoacoustic amplitude spectra for the *in vivo* median and ulnar nerves from swine I and surrounding agarose. The results for the NIR-III wavelengths [Figs. 7(b) and 7(d)] are the same as that shown in Fig. 6 with agaorose and nerve results overlapped on the same plots. At the lower NIR-II wavelengths [Figs. 7(a) and 7(c)], the nerve has similar optical properties to the mostly water-based agarose material, due to more dominance from the water absorption spectrum. As a result, distinguishing water from nerves at these NIR-II wavelengths is anticipated to be difficult. However, at the higher wavelengths, differentiation from water was possible due to the corresponding valley in the absorption spectrum of water, which can be appreciated from the agarose and PBS spectra in Fig. 3(a).

### 3.4 *Ex Vivo* Histological Assessments

**Fig. 8:**
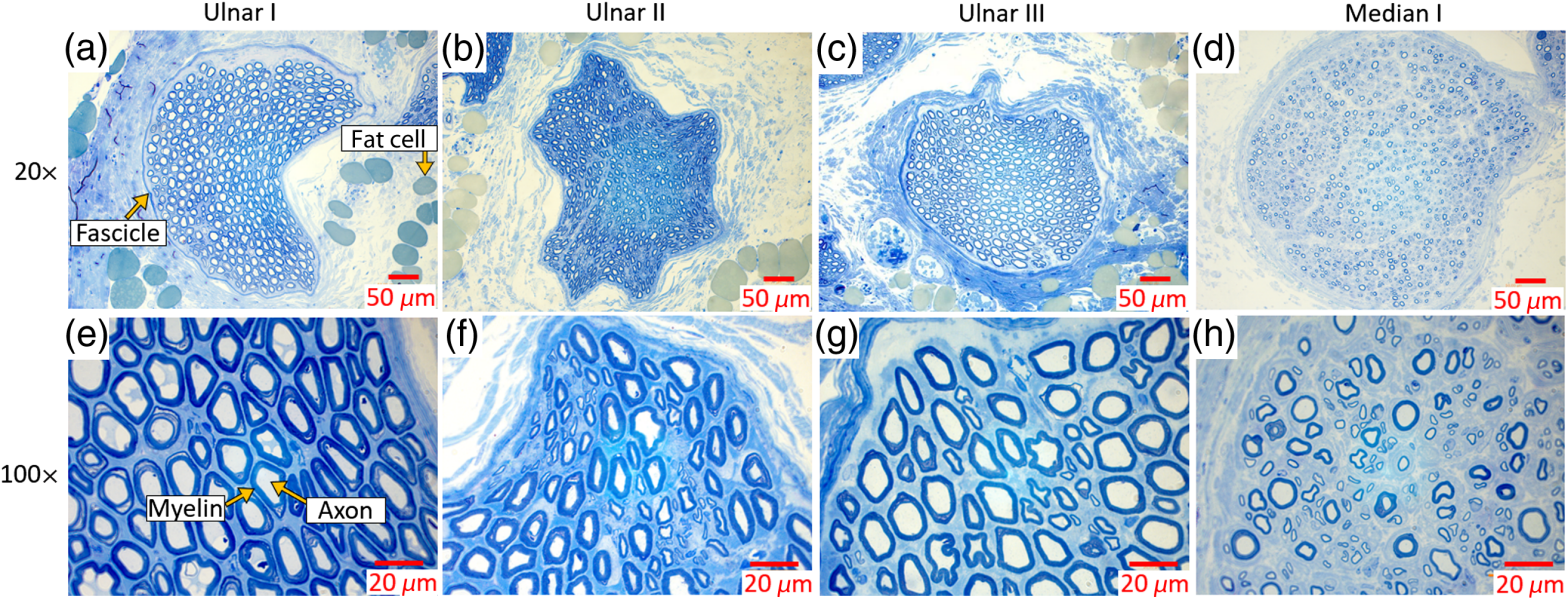
Histology sections of the ulnar nerves from (a), (e) swine I; (b), (f) swine II; (c), (g) swine III; and (d), (h) the median nerve from swine I, stained with toluidine blue to determine the presence of myelin, and visualized with (a)–(d) 20× and (e)–(h) 100× magnification.

Figure 8 shows representative histological sections of ulnar and median nerve samples from swine I. In the histological sections visualized with 20× magnification, the toluidine blue staining indicates that myelin is contained within the fascicles for each nerve sample. The staining also outlines the shape of the fascicles and demonstrates differences in fascicle shape between the nerve samples, from circular [e.g., Figs. 8(a) and 8(d)] to irregularly shaped [e.g., Figs. 8(b) and 8(c)]. In addition, fat cells are observed near the myelin-filled fascicles. The presence of fat cells indicates that both the lipid content of the fat cells and the lipid content of the myelin sheaths are the most likely contributors to the optical absorption characteristics of composite nerve tissue.

In the histological sections of Fig. 8 visualized with 100× magnification, the toluidine blue staining demonstrates the anatomy of the myelin sheath in which it encases each axon in each nerve sample. Qualitatively, axon diameter, myelin thickness, and fiber density were similar among the ulnar nerve samples. However, the regenerated median nerve has reduced axon diameter, myelin thickness, and fiber density when compared with these same properties in the ulnar nerve section.

Table 1 quantitatively compares the average nerve density, fiber diameter, myelin thickness, and G-ratio measured for all micrograph segments of each nerve sample. In particular, the average nerve density of the regenerated median nerve is greater than that of each ulnar nerve, whereas the average fiber diameter and average myelin thickness of the regenerated median nerve are lower than that of each ulnar nerve. The average G-ratio demonstrates a similar degree of myelination among the ulnar and median nerve samples.

**Table 1:** Histomorphometrics (average ± one standard deviation) of *ex vivo* ulnar and median nerve samples.

	Ulnar I	Ulnar II	Ulnar III	Median I
Nerve density ($\text{fibers}/{\mathrm{mm}}^{2}$)	4688±2755	8250±2353	6944±2837	8661±2961
Fiber diameter (μm)	14.41±4.41	12.33±2.26	13.48±3.44	9.44±2.36
Myelin thickness (μm)	5.63±1.04	5.21±1.50	5.53±0.94	3.68±0.88
G-ratio	0.59±0.105	0.58±0.095	0.58±0.094	0.60±0.067

## 4 Discussion

This work is the first to characterize the optical absorbance spectra of fresh swine nerve samples using a wide spectrum of wavelengths (i.e., 800 to 1880). In addition, *in vivo* visualization of healthy and regenerated swine nerves with multispectral photoacoustic imaging was demonstrated for the first time in the NIR-III nerve window. The absorbance of nerve tissue was unexpectedly dominated by the presence of water, as indicated by Figs. 3(a) and 3(b). However, when we implemented a possible solution to address this observation by subtracting the normalized water contribution from the normalized spectrophotometer measurements (which does not depend on known concentration ratios, as shown in the Supplementary Material), a characteristic lipid peak at 1725 nm was observed [Fig. 3(c)]. This 1725 nm peak is unique to nerve tissue as other lipids including fat^43^ and cholesterol had lower absorption peaks at 1720 and 1715 nm, respectively [Fig. 3(c)]. Similar observations of differences among the spectroscopic absorbance measurements of different lipids were previously observed between beef fat and sunflower oil^51^ and between perivascular fat and olive oil.^35^ The Supplementary Material includes additional comparisons of our results and measurement methods when incorporating soybean oil and plastisol.

We initially hypothesized that characterization of the absorbance of composite nerve tissue is advantageous for identification of nerve tissue, particularly when compared with the absorbance of other isolated lipids. This hypothesis is confirmed based on the agreement between the 1725 nm peak in the spectroscopic absorbance measurements and the 1725 nm peak in the *in vivo* photoacoustic amplitude spectra (e.g., Fig. 6). In addition, the 5 to 10 nm differences in the location of this peak in comparison with other lipids (e.g., cholesterol, murine liver fat,^43^ intramuscular goat fat,^35^ and the lipids included in the Supplementary Material) further support our hypothesis.

The comparison of the photoacoustic amplitude spectra of agarose in Fig. 6(a) and nerve in Fig. 6(b) reveals an overlap of spectral amplitudes at 1655 and 1750 nm wavelengths. This overlap is more clearly demonstrated in Figs. 7(b) and 7(d), which show the same data in a single plot for each nerve. However, the observation of the photoacoustic amplitude spectra across the entire NIR-III nerve window demonstrates the unique spectral features of nerve and agarose, including the 1725 nm absorption peak of nerve tissue. The presence or absence of these and other spectral features in multispectral photoacoustic measurements can be exploited to identify the photoacoustic signal as originating from nerve, rather than other surrounding tissue or materials in an *in vivo* setting (e.g., when implementing novel and conventional spectral unmixing approaches^59^^,^^60^). These advantages of the NIR-III window are less likely to be beneficial if trying to differentiate nerve from water at the lower NIR-II wavelengths, as demonstrated in Figs. 7(a) and 7(c).

Specific clinical applications that have the potential to benefit from the ability to identify the presence of a nerve using the 1725 nm spectral peak observed *in vivo* and confirmed with *ex vivo* spectroscopic measurements include iatrogenic nerve injury prevention, extraneural needle localization, and nerve repair surgery. In particular, nerve tissue has been differentiated from blood^31^ and tendons^29^ in previous work, albeit with lower wavelengths (e.g., 690 to 1260 nm and 1160 to 1260 nm, respectively). The combination of this previous work with the new findings presented herein supports the introduction of future clinical solutions that multiplex between lower wavelengths and the higher 1725 nm wavelength (e.g., to prevent iatrogenic nerve injury when multiple tissues are present). One may also use a combination of wavelengths that includes 1725 nm to confirm an extraneural (rather than intraneural) needle location for peripheral nerve block injections to avoid permanent nerve damage from the injection,^8^^,^^9^ which can be achieved with an optical fiber located inside the injection needle.^29^ With this setup, location information about the needle tip^23^ and distinction between different tissues^29^^,^^31^ and chromophores^59^^,^^60^ are anticipated to be possible with a combination of ultrasound and photoacoustic imaging.^32^^,^^33^^,^^38^^,^^61^ Robotic approaches may additionally be introduced to minimize operator dependence.^23^^,^^62^ A third possible clinical implementation is to expose a nerve during plastic or reconstructive surgery (e.g., during nerve repair) to ensure that we only operate on nerves needing repair, rather than healthy or regenerating nerves. Depending on the clinical application and surrounding environment, lower wavelengths can be used when nerves are surrounded by other tissues, whereas higher wavelengths have the potential to offer greater photoacoustic amplitude-related feature detection when nerves are isolated [see Fig. 3(a)]. As the intended focus of this paper is optical absorbance characterization, wavelength selection, and corresponding demonstrations of the feasibility of *in vivo* photoacoustic imaging of isolated nerves, our contributions successfully establish a new scientific foundation to be leveraged by future clinical applications.

Histology confirmed that the likely source of the 1725 nm absorption peak was the presence of lipids within the myelin sheath and fat cells of nerve tissue (Fig. 8). Histomorphmetrics reveal overlapping mean ± standard deviation nerve fiber density and G-ratio (i.e., degree of myelination) between the regenerated median nerve samples and the control ulnar nerve samples (Table 1). Comparisons across spectroscopic absorbance measurements and photoacoustic amplitude spectra similarly revealed negligible optical absorption changes between the control (i.e., ulnar) and regenerated (i.e., median) nerve samples. Given this similarity, our newly introduced nerve optical absorption characterization results may be employed as a future reference spectrum for photoacoustic imaging of other similarly myelinated nerves (i.e., other than healthy ulnar and regenerated median nerves) and may also be utilized for other optics-based nerve imaging techniques (e.g., diffuse reflectance spectroscopy^63^). As with any optics-based application, there are also possible cases in which the information provided by the 1725 nm peak may not be useful (e.g., if there is significant spectral overlap with other chromophores of interest, as observed in Fig. 7 when using NIR-II wavelengths).

As noted above, purely optical imaging methods have the potential to utilize the new optical characterizations presented herein.^63^ The benefits of photoacoustic imaging relative to purely optical imaging methods include better penetration depth and spatial resolution.^64^ Although the optical penetration depth in Fig. 4 appears to be ∼1 to 2 mm from the surface of the nerve when illuminated with a wavelength of 1725 nm and a mean fluence of $∼18\;\mathrm{mJ}/{\mathrm{cm}}^{2}$, the ANSI limit for a wavelength of 1725 nm is $1\;J/{\mathrm{cm}}^{2}$ when imaging through skin.^65^ Therefore, higher energies are likely to achieve greater penetration depths than that presented in Fig. 4, and the specific laser safety limit for exposed nerve tissue remains to be determined.

Despite the agreement between the optical absorption peak of nerves at 1725 nm in the spectroscopic absorbance measurements and photoacoustic amplitude spectra [Fig. 6(b)], two discrepancies were observed between the datasets. First, although absorbance measurements of nerve tissue were dominated by water absorption [Fig. 3(b)], photoacoustic amplitude spectra measurements of nerve tissue were dominated by lipid absorption [Fig. 6(b)]. A possible reason for this discrepancy is the presence of excess PBS on and surrounding the nerve in the sample holder due to the nerve being transported from the operating room to the spectroscopy machine in a container filled with PBS. Second, the amplitude of the spectroscopic absorbance measurements of nerve tissue in the range of 1745 to 1765 nm is greater than that of the corresponding normalized photoacoustic amplitude spectra [Fig. 6(b)]. Temperature differences between the *ex vivo* and *in vivo* nerve samples are one potential cause of this difference, particularly when considering that the optical absorption of chromophores such as water can vary with temperature.^51^ Nonetheless, the observed differences do not affect the major conclusions of our study as we are most interested in peak values, which are consistent across both spectrophotometer and photoacoustic measurements (and are also consistent with the spectrophotometer results obtained from a myelinated phrenic nerve sample, as shown in the Supplementary Material).

Future work will be dedicated to additional technology development, such as the implementation of improved light delivery designs^66^ and photoacoustic-based diagnosis of nerve health (e.g., degree of myelination). In particular, the experimental procedures implemented to isolate *in vivo* nerves are similar to the surgical approach used to expose and treat nerve injuries. Therefore, the proposed approach is promising for the development of new technology catered to nerve imaging during nerve repair surgeries within the NIR-III nerve window that has now been characterized for the first time, to the authors’ knowledge.

## 5 Conclusion

The work presented in this paper provides foundational evidence to support an optimized *in vivo* multispectral photoacoustic nerve imaging approach using NIR-III wavelengths. Spectroscopic and photoacoustic imaging analyses demonstrated that exploiting the 1725 nm absorption peak of nerve tissue enables the identification of nerve tissue and the differentiation of nerve tissue from other substances, such as aqueous materials (e.g., our custom agarose block). Although histology identified structural differences between the regenerated and control nerve samples (e.g., fiber diameter, myelin thickness), the combination of spectroscopic, photoacoustic imaging, and histomorphmetric G-ratio (i.e., degree of myelination) analyses revealed negligible differences between the regenerated and control nerve samples due to the similar presence of lipids within each myelinated nerve. These results highlight the clinical promise of multispectral photoacoustic imaging as an intraoperative technique to determine the presence of, and prevent iatrogenic injury to, myelinated nerves, with possible future implications for other optics-based technologies.

## Code, Data, and Materials Availability

Unrestricted code and data are available upon reasonable request to the corresponding author.
