# Supplementary material for: Optical absorption spectra and corresponding in vivo photoacoustic visualization of exposed peripheral nerves
Source: J Biomed Opt. 2023 Sep 4;28(9):097001. doi: 10.1117/1.JBO.28.9.097001 (PMC10475953; doi:10.1117/1.JBO.28.9.097001)
Supplement: Supplementary file 1 [file JBO_028_097001_SD001.pdf]

Optical Absorption Spectra and Corresponding *In Vivo* Photoacoustic Visualization of Exposed Peripheral Nerves: Supplementary Material

Michelle T. Graham[a], Arunima Sharma[a], William M. Padovano[b], Visakha Suresh[b], Arlene Chiu[a], Susanna M. Thon[a], Sami Tuffaha[b], Muyinatu A. Lediju Bell[a,c,d]
[a]Department of Electrical and Computer Engineering, Johns Hopkins University, Baltimore, MD, USA
[b]Department of Plastic and Reconstructive Surgery, Johns Hopkins School of Medicine, Baltimore, MD, USA
[c]Department of Biomedical Engineering, Johns Hopkins University, Baltimore, MD, USA
[d]Department of Computer Science, Johns Hopkins University, Baltimore, MD, USA

1 Normalized Subtraction of Spectrophotometer Measurements

To account for the unexpected contribution of PBS in Fig. 3, each absorbance spectra was normalized and scaled from 0 to 1, then the normalized and scaled PBS absorbance spectrum was subtracted from each normalized and scaled nerve absorbance spectra measurement. The final result was then rescaled for display in Fig. 3(c). These details are described in Section 3.1.

The rationale supporting this implementation relies on three assumptions to recover local optical absorption peaks associated with a single chromophore component in a mixture of chromophore components:

1. One component is more similar to the mixture than the mixture is to the component(s) being recovered.
2. The peak of the absorption spectrum of one component occurs in the valley of the absorption spectrum of other components.
3. The peaks of the absorption spectra of the components in the mixture occur at different wavelengths.

The first assumption is true for the nerve results because the spectrum of water is generally an order of magnitude greater than that of fat, particularly when comparing peak optical absorption values.[43] Therefore, the PBS spectrum in Fig. 3(a) is more similar to the spectra obtained with nerves surrounded by PBS, when compared to the similarity between the combined spectra (i.e., nerve and PBS) and the lipid spectra in Fig. 3(a). The second assumption is true for PBS relative to cholesterol (i.e., the primary lipid in the myelin sheath of nerves[52–55]) in Fig. 3. The third assumption is true for the PBS and lipid spectra within the NIR-III nerve window in Fig. 3.

Because these three assumptions are true, yet the ratio of PBS-to-lipid is unknown, the absorbance was normalized to decrease the effects of the ratio of PBS-to-lipid. We understand that knowledge of this ratio would otherwise impact the absorbance (e.g., a higher concentration ratio will yield a higher absorbance across the spectrum), but the shape of the spectral peak is expected to remain the same (e.g., when absorbance is calculated for different concentration ratios, the normalized results are expected to be similar). Therefore, by normalizing and scaling, we decrease the effects of concentration, and in this case knowledge of the PBS-to-lipid ratio is not required to implement the subtraction method described above and in Section 3.1.

To mathematically describe our logic and implement *in silico* validation experiments, the known absorption profile of water is μ_{water}, and the known absorption profile of fat is μ_{fat}, with

various combinations of water and fat concentrations (i.e., factors of α_1 and α_2, respectively) mathematically described as a linear combination:

$$C(\alpha_1, \alpha_2) = \alpha_1 \mu_{water} + \alpha_2 \mu_{fat} \tag{1}$$

When α_1 is varied to equal 0.1, 1, or 10, while $\alpha_2 = 1$, and μ_{water} is normalized from 0 to 1 then subtracted from the normalized values of $C(\alpha_1, \alpha_2)$, we achieve the results in Fig. S1, which each produce a peak that consistently matches the primary of peak of μ_{fat} at 1715 nm wavelength. There are similarly matching peaks at 1205 and 2135 nm wavelengths in Fig. S1(d). Otherwise, the dissimilarities between the resultant subtracted spectrum and the lipid spectrum at wavelengths spanning 1400-1630 nm and 1885-2090 nm occur because there is no local peak to recover (at 1885-2090 nm wavelengths) or the second assumption described above was not met (at 1400-1630 nm wavelengths).

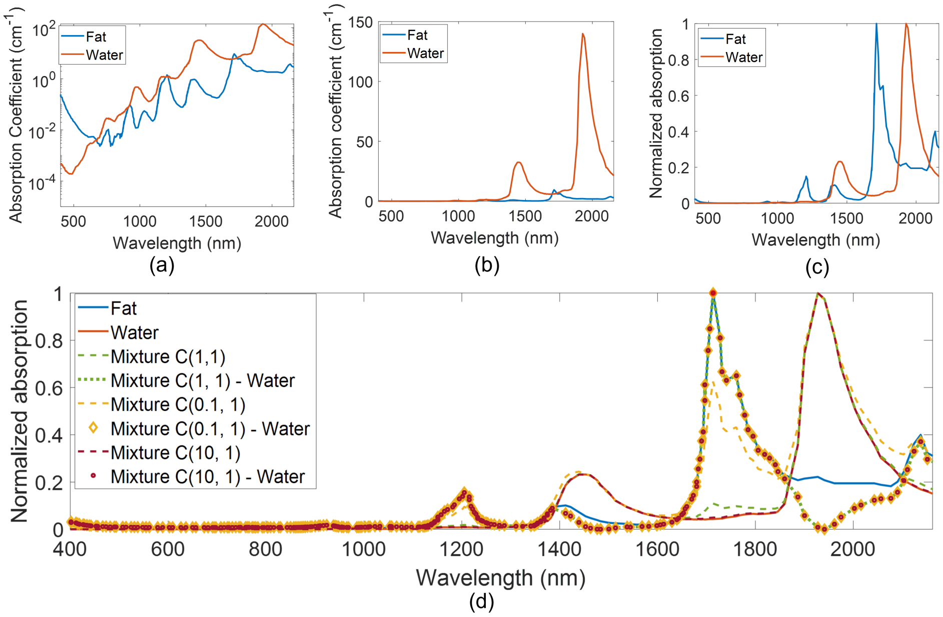

Fig S1 Validation of subtracting normalized and scaled PBS absorbance from normalized and scaled nerve absorbance spectra using *in silico* experiments. The absorption spectra of fat (i.e., a lipid) and water were extracted from[43] and are shown on a (a) log scale, (b) linear scale, and (c) normalized scale. (d) The normalized and scaled absorbance of fat and water are shown with normalized and scaled concentration mixtures described by α_1= 0.1, 1, or 10 and α_2 = 1, based on Eq. (1). The normalized and scaled water spectrum was subtracted from the normalized and scaled mixture spectra, yielding similar results to fat.

2 Differences in the Optical Absorption of Lipids and Nerves (including a Phrenic Nerve)

To provide additional confirmation of the differences in absorption peaks between lipids and nerves, Fig. S2 shows results obtained after augmenting Fig. 3 in the manuscript with additional analyzed lipid samples and a myelinated phrenic nerve sample (included for demonstration, although not a peripheral nerve). We used the methods reported in Section 2.2 to optically characterize multiple lipids (i.e., cholesterol, soybean oil, and plastisol) in multiple formats (including solid format for plastisol, liquid format for soybean oil, and powdered format for cholesterol) and to optically characterize the myelinated left phrenic nerve sample dissected from near the diaphragm

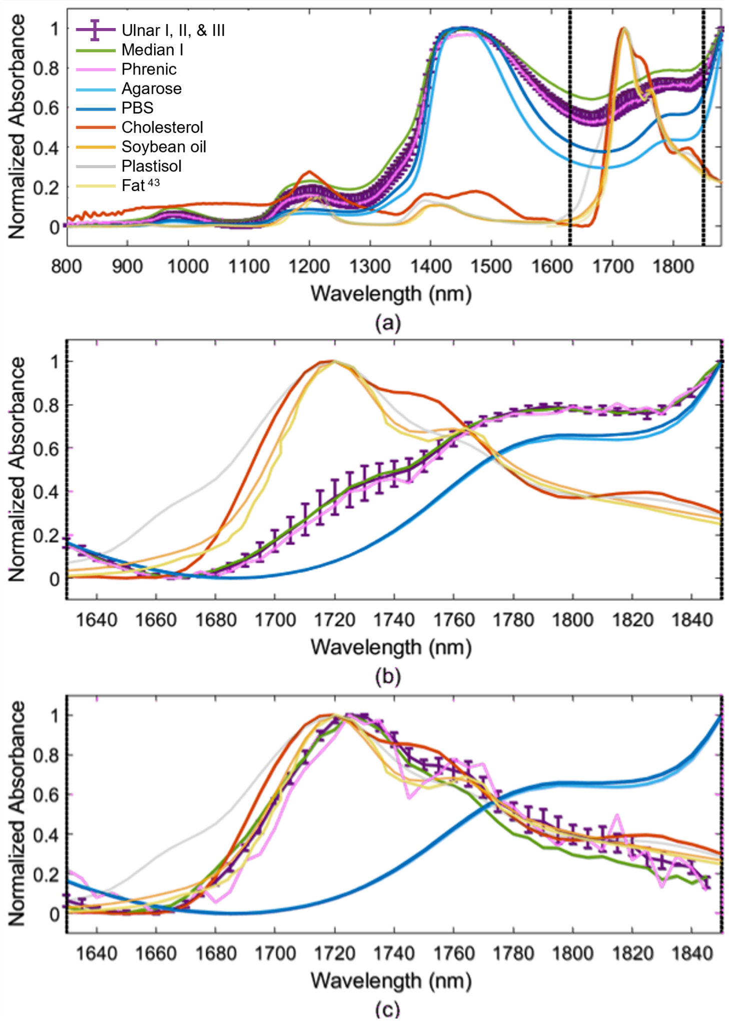

Fig S2 Absorbance measurements of three ulnar nerve samples (mean ± one standard deviation), one median nerve sample, one phrenic nerve sample, and samples of powdered cholesterol, 3% w/v agarose, phosphate buffered saline (PBS), soybean oil, and plastisol, shown for (a) 800-1880 nm wavelengths, (b) 1630-1850 nm wavelengths (indicated by vertical dashed lines in panels (a)-(c)), and (c) 1630-1850 nm wavelengths after the PBS absorbance spectrum was subtracted from each nerve measurement. In each case, the normalized optical absorption of fat was extracted from Ref.[43] and is shown for comparison.

of a swine (with the epineurium intact) for comparison with the peripheral nerve samples (characterized after epineurectomy). We consistently achieved similar lipid absorption peaks for fat,[43] cholesterol, soybean oil, and plastisol. In addition, the lipid absorption peak consistently differed from the 1725 nm peak observed for nerves, including the phrenic nerve.

It is additionally promising that after repeated measurements on multiple nerves, we achieved the same result regarding the location of the 1725 nm peak. As the focus of our manuscript is nerve characterization, these nerves were characterized in a state that was as close to the *in vivo* state as possible. Any possible environmental influences that may affect the multiple lipid reference points presented above ultimately do not affect our major conclusions about nerve characterization. Therefore, the major conclusions of our manuscript are supported by the presented nerve characterization results.
